# Brain sodium sensing for regulation of thirst, salt appetite, and blood pressure

**DOI:** 10.14814/phy2.15970

**Published:** 2024-03-13

**Authors:** Takeshi Y. Hiyama

**Affiliations:** ^1^ Department of Integrative Physiology Tottori University Graduate School and Faculty of Medicine Yonago Japan

**Keywords:** blood pressure, Na_x_, OVLT, salt appetite, subfornical organ, thirst

## Abstract

The brain possesses intricate mechanisms for monitoring sodium (Na) levels in body fluids. During prolonged dehydration, the brain detects variations in body fluids and produces sensations of thirst and aversions to salty tastes. At the core of these processes Na_x_, the brain's Na sensor, exists. Specialized neural nuclei, namely the subfornical organ (SFO) and organum vasculosum of the lamina terminalis (OVLT), which lack the blood–brain barrier, play pivotal roles. Within the glia enveloping the neurons in these regions, Na_x_ collaborates with Na^+^/K^+^‐ATPase and glycolytic enzymes to drive glycolysis in response to elevated Na levels. Lactate released from these glia cells activates nearby inhibitory neurons. The SFO hosts distinct types of angiotensin II‐sensitive neurons encoding thirst and salt appetite, respectively. During dehydration, Na_x_‐activated inhibitory neurons suppress salt‐appetite neuron's activity, whereas salt deficiency reduces thirst neuron's activity through cholecystokinin. Prolonged dehydration increases the Na sensitivity of Na_x_ via increased endothelin expression in the SFO. So far, patients with essential hypernatremia have been reported to lose thirst and antidiuretic hormone release due to Na_x_‐targeting autoantibodies. Inflammation in the SFO underlies the symptoms. Furthermore, Na_x_ activation in the OVLT, driven by Na retention, stimulates the sympathetic nervous system via acid‐sensing ion channels, contributing to a blood pressure elevation.

## 1 INTRODUCTION

The Na^+^ concentration ([Na^+^]) in human blood and cerebrospinal fluid (CSF) is controlled within the range of 135–145 mEq/L (Peruzzo et al., 2010). This variation arises from interindividual differences, as sodium concentrations are maintained strictly constant within each individual. To achieve this, the brain needs to continuously monitor blood [Na^+^] in specialized brain nuclei, which are exposed to both circulating blood and CSF. In this review, we provide an overview of the Na^+^‐sensing mechanisms in the brain from a physiological perspective, elucidating their roles and functions.

## 2 Na_X_: A Na SENSOR IN SENSORY CIRCUMVENTRICULAR ORGANS

**FIGURE 1:**
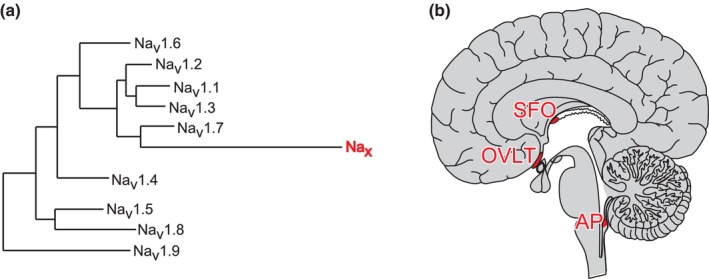
Na_x_ channel and its expression loci in the brain. (a) Phylogenetic tree of voltage‐gated Na channel α subunits. (b) Location of sensory circumventricular organs (red) in a sagittal view of human brains. Reproduced with minor modifications from Goldin et al. (2000) for (a).

Na_x_ is a Na channel, which was cloned from a cDNA library derived from cultured rat astrocytes (Gautron et al., 1992). Na_x_ was originally classified as a subfamily of voltage‐gated Na channels (Figure 1a; in humans, Na_x_ is encoded by the *SCN7A* gene; Noda & Hiyama, 2005, 2015a, 2015b). However, attempts to detect voltage‐dependent currents from heterologously expressed Na_x_ failed due to the distinctive regions essential for voltage sensing and inactivation, which differ from other Na_v_ channel family members (Goldin et al., 2000; Hiyama & Noda, 2016; Noda & Hiyama, 2015b).

Expression of Na_x_ can be observed in specific brain regions, including the subfornical organ (SFO) and organum vasculosum of the lamina terminalis (OVLT) (Figure 1b; Watanabe et al., 2000). Cells immunopositive for Na_x_ in these areas are glial fibrillary acidic protein (GFAP)‐positive glia, specifically astrocytes and ependymal cells (Watanabe et al., 2006).

The SFO and OVLT, along with the area postrema (AP), are collectively termed sensory circumventricular organs (sensory CVOs) (Lind, 1987). These regions are unique to the brain because of the absence of a blood–brain barrier (BBB), which contains neuronal cell bodies and extensive afferent and efferent neural connections with many other brain nuclei (Johnson & Gross, 1993). Furthermore, the expression of various hormone receptors, including angiotensin II receptors, has been reported in sensory CVOs (McKinley et al., 2003; Premer et al., 2013; Song et al., 1992). Accordingly, sensory CVOs are sites where the brain continuously monitors circulating substances to determine the bodily status.

## 3 Na_X_ AND SALT PREFERENCE

To investigate physiological role of Na_x_ in vivo, *Na*_*x*_‐knockout (*Na*_*x*_‐KO) mice were generated (Watanabe et al., 2000). To assess the preference for salt, mice were provided with pure water and a 0.3 M NaCl solution, and the salt intake amounts were compared between *Na*_*x*_‐KO and wild‐type (WT) mice as a ratio to total fluid intake. When the mice were sufficiently hydrated with water, neither *Na*_*x*_‐KO nor WT mice exhibited specific preferences (Hiyama et al., 2004; Watanabe et al., 2000). However, under dehydrated conditions, WT mice displayed extensive water intake and an aversion to salty water, whereas *Na*_*x*_‐KO mice did not exhibit such aversion.

Electrophysiological analyses of the taste sensory neural pathways from the tongue confirmed that *Na*_*x*_‐KO mice possess normal taste sensitivity to Na^+^ (Watanabe et al., 2000). Therefore, the behavioral abnormalities observed in *Na*_*x*_‐KO mice can be attributed to internal sensing mechanisms that detect [Na^+^] in body fluids. Consistently, intracerebroventricular infusion of a hypertonic Na^+^ solution induced salt aversion in WT mice, but not in *Na*_*x*_‐KO mice (Hiyama et al., 2004). Such aversion was not elicited by intracerebroventricular infusion of mannitol solution with equivalent osmolality in either mouse genotype. Thus, although Na_x_ is involved in [Na^+^] sensing, it does not participate in osmotic pressure sensing within the brain.

Following water restriction, neuronal activity in the SFO and OVLT of *Na*_*x*_‐KO mice was significantly higher than that in WT mice, as estimated by Fos immunoreactivity, (Watanabe et al., 2000). Site‐specific reconstitution of the *Na*_*x*_ gene completely restored the abnormal salt intake behavior in *Na*_*x*_‐KO mice. These results highlight the SFO as a crucial site for brain [Na^+^] sensing in regulating salt intake behavior and emphasize the vital role of Na_x_ in this sensing mechanism.

We tested whether Na_x_ responds to changes in extracellular [Na^+^] ([Na^+^]_o_) and functions as a [Na^+^]_o_ sensor within the brain. This was confirmed by imaging analyses of intracellular [Na^+^] changes when [Na^+^]_o_ is gradually increased from the control level (145 mM) (Hiyama et al., 2002). A series of solutions with several [Na^+^] exceeding the control level was applied to Na_x_‐positive cells isolated from the SFO, resulting in a small, but sustained Na^+^ influx (Hiyama et al., 2002). The threshold value of Na_x_ for [Na^+^]_o_ was approximately 150 mM. These [Na^+^]_o_‐sensitive cells were insensitive to changes in osmotic pressure or [Cl^−^]_o_ increase (Hiyama et al., 2002). As expected, SFO cells derived from *Na*_*x*_‐KO mice do not exhibit these responses, and the introduction of Na_x_ cDNA restores [Na^+^]_o_ sensitivity to cells from *Na*_*x*_‐KO mice (Noda & Hiyama, 2005).

**FIGURE 2:**
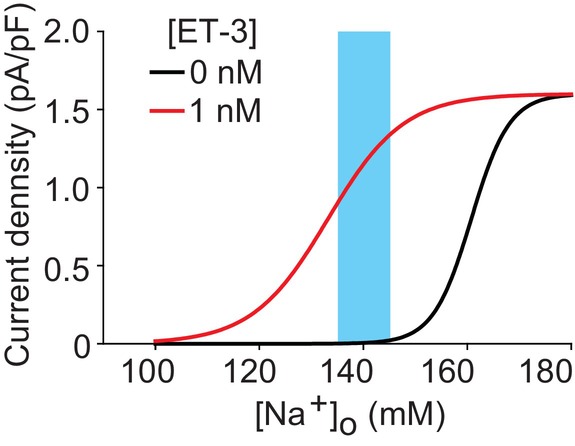
Relationship between current density (current amplitude/cell capacitance) and [Na^+^]_o_ in the presence or absence of ET‐3 (1 nM). Blue area shows the normal range of blood [Na^+^]. This variability is due to individual differences, and the sodium levels of each individual are strictly maintained.

Functional analyses of cell lines heterologously expressing Na_x_ showed that the [Na^+^]_o_ sensitivity of Na_x_ within these cells is similar to that of SFO glia (Matsumoto et al., 2015). The cell‐line experiments revealed that the cation selectivity order of the Na_x_ channel was Na^+^ ≈ Li^+^ > Rb^+^ > Cs^+^. These results further support our notion that Na_x_ itself functions as an Na channel that is sensitive to increases in [Na^+^]_o_. Subsequent research revealed the presence of a dynamic regulatory mechanism for Na_x_ sensitivity in the SFO. During dehydration, the local expression of the vasorelaxation hormone endothelin‐3 (ET‐3) increases. Endothelin receptor B (ET_B_R) is predominantly expressed in the glia of the brain (Hori et al., 1992) and is highly expressed in the SFO (Hindmarch et al., 2008). Pharmacological experiments revealed that ET_B_R signaling is involved in the modulation of Na_x_ threshold through protein kinase C and ERK1/2 activation (Hiyama et al., 2013). This mechanism leads to an enhancement of the body fluid [Na^+^] sensor activation (Figure 2).

## 4 LACTATE SIGNALING FROM GLIAS TO NEURONS

Na_x_ channels are expressed in the fine processes of astrocytes and ependymal cells, which envelope a specific subpopulation of GABAergic neurons within the SFO (Watanabe et al., 2006). When tested with cells isolated from the SFO, Na_x_‐positive glia are sensitive to increases in [Na^+^]_o_, whereas SFO neurons are nonresponsive (Hiyama et al., 2002; Watanabe et al., 2006). Neural activity in the SFO is modulated in an Na_x_‐dependent manner, indicating the presence of a signaling mechanism from Na_x_‐positive glia to neighboring neurons (Watanabe et al., 2000).

**FIGURE 3:**
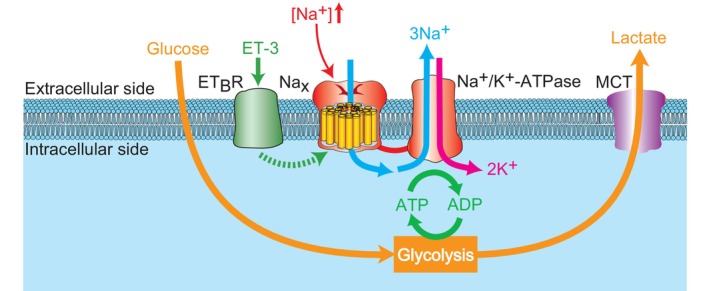
[Na^+^]‐sensing mechanism and Na_x_‐dependent control of lactate production in the glia (ependymal cells and astrocytes) in the subfornical organ (SFO)/organum vasculosum of the lamina terminalis (OVLT). When [Na^+^]_o_ surpasses a threshold, Na_x_ channels open, triggering Na^+^/K^+^‐ATPase activation and accelerating ATP consumption. To meet ATP demands, anaerobic glycolysis is boosted in glia, enhancing glucose uptake. The glia release lactate, the glycolysis end product. ET‐3, endothelin‐3; ET_B_R, endothelin receptor type B. Modified from Shimizu et al. (2007).

To gain a better understanding of the physiological processes involving Na_x_ in glia, yeast two‐hybrid screening was conducted to identify molecules that interact with the cytoplasmic domain of Na_x_. Na_x_ channels were found to stably bind to the α1 and α2 subunits of Na^+^/K^+^‐ATPase through their carboxyl‐terminal regions (Shimizu et al., 2007). The carboxyl‐terminal domain of Na_x_ binds to nearby the catalytic region of the Na^+^/K^+^‐ATPase (Shimizu et al., 2007) (Figure 3). Subsequent analyses revealed a close functional coupling between Na_x_ and Na^+^/K^+^‐ATPase. [Na^+^]_o_‐dependent activation of Na_x_ enhances glucose uptake within the same cells, a process inhibited by the Na^+^/K^+^‐ATPase inhibitor ouabain (Shimizu et al., 2007). Conversely, the induction of Na^+^ influx with an ionophore alone failed to replicate glucose uptake, highlighting the essential role of the carboxyl‐terminal region of Na_x_. The yeast two‐hybrid screening also identified triosephosphate isomerase (TPI), an enzyme involved in the anaerobic glycolysis pathway, as a candidate protein that interacts with the carboxyl‐terminal region of Na_x_ (Shimizu et al., 2007). The localization of the glycolytic enzyme complex containing TPI near the catalytic region of ATPase through the Na_x_ carboxyl‐terminal region may facilitate an effective ATP supply, leading to the activation of anaerobic glycolysis following Na_x_ activation.

The activation of anaerobic glycolysis in glia leads to the generation and release of lactate (Walz & Mukerji, 1988). Incubation with high‐Na solutions results in increased lactate release from the SFO tissues of WT mice, but not in *Na*_*x*_‐KO mice (Shimizu et al., 2007). Therefore, it is suggested that the [Na^+^]_o_ information is converted into the amount of lactate released from Na_x_‐positive glia.

**FIGURE 4:**
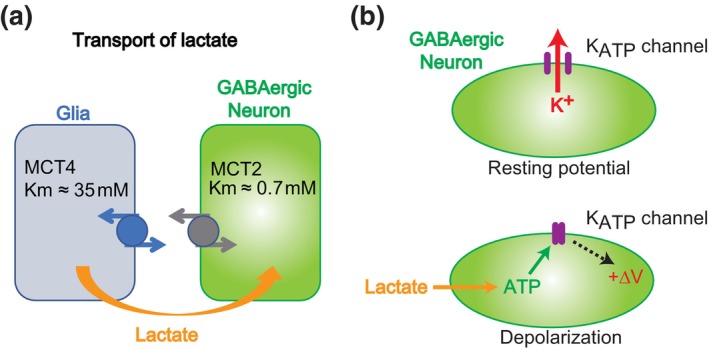
Lactate signaling by glia to control neighboring inhibitory neurons. (a) Schematic outlining explaining lactate transport from glia to neurons. (b) Depolarization mechanism following the lactate transport. MCT, monocarboxylate transporter.

Lactate, the end product of glycolysis in astrocytes, is vital for neuronal oxidative ATP production (Brooks, 2009; Magistretti et al., 1999; Schurr et al., 1999; Wiesinger et al., 1997). Intercellular lactate transport is mediated by monocarboxylate transporters (MCTs). Astrocytes express MCT4 (Km = 35 mM), a low‐affinity transporter implicated in the transport of lactate generated through glycolysis (Pierre & Pellerin, 2005). In contrast, neurons express MCT2 (Km = 0.7 mM), a high‐affinity transporter suitable for extracellular lactate uptake. Consequently, astrocyte‐produced lactate is taken up and oxidized by neighboring neurons (Figure 4a; Magistretti et al., 1999).

Electrophysiological experiments using tissue slices revealed the spontaneous firing of the neighboring GABAergic neurons in the SFO. When exposed to high‐[Na^+^] solutions, the firing rate of the GABAergic neurons in the SFO of WT mice gradually increases (Shimizu et al., 2007). In contrast, the activity of GABAergic neurons in the SFO of *Na*_*x*_‐KO mice remains unchanged even after the [Na^+^]_o_ elevation. The firing of GABAergic neurons in both genotypes increases upon the direct addition of lactate (Shimizu et al., 2007). MCT inhibition suppresses the [Na^+^]_o_‐dependent enhancement of GABAergic firing (Shimizu et al., 2007).

Further pharmacological analyses revealed that the fundamental mechanism of this activation involves changes in the membrane potential of GABAergic neurons. With an increase in intracellular ATP levels resulting from ATP hydrolysis, ATP‐sensitive K^+^ channels (Kir6.2/K_ATP_ channels) close (Shimizu et al., 2007). The closure of K_ATP_ channels leads to membrane depolarization in GABAergic neurons (Figure 4b). Thus, lactate released from astrocytes serves as an energy supply source, elevating the firing activity of these GABAergic neurons in a [Na^+^]_o_‐dependent manner.

## 5 ANGIOTENSIN II‐SENSITIVE THIRST AND SALT‐APPETITE NEURONS

**FIGURE 5:**
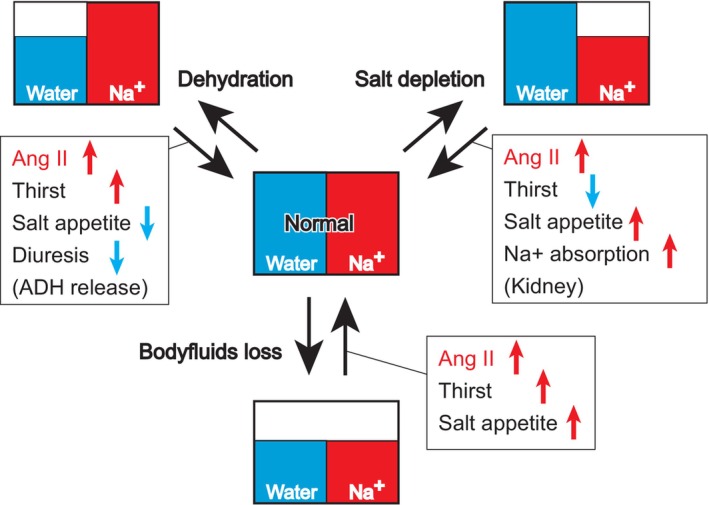
Na/water balance in body fluids and physiological responses. Blood angiotensin II levels increase under any of the three conditions. The physiological responses in each square contribute to homeostatic recovery from each condition.

To understand the relationship between the local signaling of Na sensors in the SFO and salt‐appetite behavior, as suggested by *Na*_*x*_‐KO mouse studies, a deeper understanding of the neural circuits within the brain that regulate fluid homeostasis is required. Previous findings have suggested a connection between angiotensin II and the control of thirst and salt appetite; intracranial injections of angiotensin II stimulate both water and salt intake (Buggy & Fisher, 1974; Fitzsimons, 1998; Rowland et al., 1996). After inducing thirst (water deprivation), body fluid loss (water and sodium depletion), and salt deficiency (sodium depletion) in mice, angiotensin II levels in the bloodstream are increased to similar levels under all three conditions (Figure 5; Matsuda et al., 2017).

**FIGURE 6:**
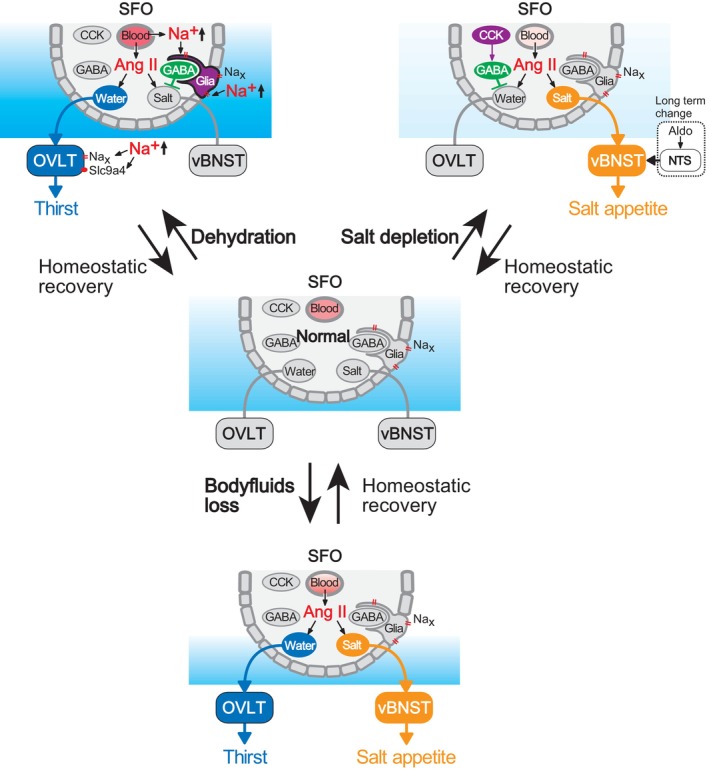
Regulation of thirst and salt appetite from the subfornical organ (SFO). The SFO contains neuronal cell bodies and fenestrated capillaries allowing angiotensin II (Ang II) entry. Under dehydrated conditions (top, left), Ang II stimulates both SFO(→ventral bed nucleus of the stria terminalis, vBNST) neurons (for salt appetite) and SFO(→organum vasculosum of the lamina terminalis, OVLT) neurons (for thirst). However, Na_x_ channels in glia respond to increased [Na^+^]_o_, activating lactate signaling, leading to activation of GABAergic neurons and suppression of SFO(→vBNST) neurons. In Na‐depleted conditions (top, right), although Ang II stimulates both types, cholecystokinin (CCK) neurons in the SFO are activated and suppress the activities of SFO(→OVLT) neurons via GABAergic activation, inhibiting water intake. Ang II successfully activates only SFO(→vBNST) neurons, enhancing salt appetite. Salt depletion triggers aldosterone release, which affects the gene expression profiles in the nucleus of the solitary tract (NTS) neurons. Consequently, enhancement of the neuronal activity occurs, and salt appetite is induced. Under both water and Na depletion (bottom), Ang II activates both types of neurons in the absence of Na_x_ or CCK signals. Modified from Matsuda et al. (2017).

In mice, the angiotensin type 1a receptor (AT1a) has been shown to play a major role in angiotensin II‐dependent thirst and salt appetite (Figure 6; Matsuda et al., 2017). During dehydration and salt deficiency, AT1a‐expressing neurons in the SFO are predominantly activated. Mice lacking AT1a expression in the SFO exhibit significantly reduced water and salt intakes during water‐/Na‐deficient states (with almost no salt intake). Further detailed analyses have revealed the existence of two types of AT1a expressing neurons in the SFO: neurons extending neural processes to the OVLT, which are referred to as SFO(→OVLT) neurons, and those projecting to the ventral bed nucleus of the stria terminalis (vBNST), which are referred to as SFO(→vBNST) neurons. Mice with *AT1a* gene deletion only in neurons with neural connections to the OVLT exhibit reduced drinking volumes during water/Na deficiency. Conversely, mice with AT1a loss in neurons with neural connections to the vBNST show decreased salt intake.

We used optogenetics, which is a technique for controlling neural activity using light, to selectively manipulate thirst and salt appetite in mice (Matsuda et al., 2017). Activation of SFO(→OVLT) neurons induced drinking behavior when stimulated by light, while inhibition reduced drinking volume in dehydrated mice. Similarly, activation of SFO(→vBNST) neurons increases salt intake in dehydrated mice, which typically avoid salt, while inhibition suppresses salt intake in salt‐deficient mice.

## 6 STATE‐DEPENDENT SUPPRESSION OF THIRST OR SALT APPETITE

During dehydration, when salt intake is suppressed despite elevated angiotensin II levels, the Na_x_ sensor in the glia of the SFO is activated, leading to the activation of inhibitory neurons through lactate. Under conditions of high [Na^+^]_o_, the activity of SFO(→vBNST) neurons is suppressed by inhibitory neurons, which are activated by Na_x_ (Matsuda et al., 2017).

In contrast, during salt deficiency (thirst is suppressed despite an increase in angiotensin II), cholecystokinin (CCK) is released in the SFO, which has inhibitory effects on water intake (Matsuda et al., 2020). Examination of neural activity within the SFO revealed the presence of a group of inhibitory neurons suppressing the activity of SFO(→OVLT) neurons. CCK‐producing excitatory neurons in the SFO stimulate the activity of the inhibitory GABAergic interneurons via CCK‐B receptors.

In ancient times, animals still lived in the oceans, where they had constant access to water and salt. In such environments, angiotensin II likely evolved as a signal that promotes salt and water intake when body fluids are lost. Evolutionarily, as organisms transitioned into terrestrial habitats, they experienced prolonged periods of water and salt deprivation. This evolutionary history might underlie the complex mechanisms whereby Na_x_‐positive glia or CCK‐producing neurons are activated by an imbalance of water and Na^+^ to regulate the water/salt appetite by angiotensin II.

## 7 REGULATORY MECHANISMS OF SALT APPETITE SIGNALS

Long‐term changes in salt appetite may also affected by a population of neurons in the nucleus of the solitary tract (NTS) that expresses both the mineralocorticoid receptor and the enzyme 11β‐hydroxysteroid dehydrogenase type 2 (HSD2) (Jarvie & Palmiter, 2017). HSD2 neurons are not directly activated by aldosterone itself. However, a prolonged low‐sodium diet alters the expression of voltage‐gated channels via aldosterone, leading to spontaneous firing (Resch et al., 2017). HSD2 neurons do not induce salt appetite when activated independently of other cells, and the suppression of their activity does not completely eliminate salt appetite (Jarvie & Palmiter, 2017; Resch et al., 2017). Nevertheless, activation after angiotensin II administration induces salt appetite, suggesting a long‐term role of aldosterone in the modulation of the angiotensin‐activated salt appetite. In contrast, neurons expressing oxytocin receptors in the lateral parabrachial nucleus (LPBN), which receive input from the paraventricular nucleus (PVN), reportedly suppress salt appetite when activated (Ryan et al., 2017). Both HSD2 neurons and LPBN oxytocin receptor‐expressing neurons have neural connections with the vBNST, the target of salt‐appetite neurons in the SFO, suggesting the conversion of salt‐appetite signals in the vBNST and the existence of some regulatory neural mechanisms in this nucleus.

## 8 Na_X_ IN THIRST CONTROLS

**FIGURE 7:**
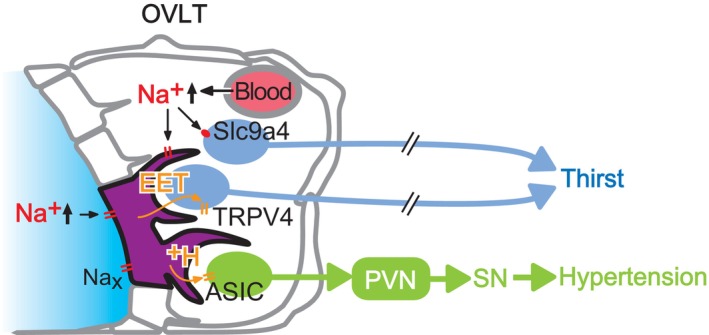
Na^+^ sensing in the organum vasculosum of the lamina terminalis (OVLT) and regulation of thirst and blood pressure. Increased [Na^+^]_o_ activates Slc9a4‐positive neurons directly to induce thirst. Na_x_ channels in glias (ependymal cells and astrocytes) are also activated in responding to [Na^+^]_o_. This activation leads to 5,6‐ epoxyeicosatrienoic acid (EET) synthesis. Released EETs activate TRPV4 channels of neighboring neurons, which potentially controls water intake behavior. Na_x_ activation also induces H^+^ (with lactic acid ions) release from Na_x_‐expressing ependymal cells and astrocytes through monocarboxylate transporters (MCTs). The resultant extracellular acidification stimulates OVLT(→paraventricular nucleus, PVN) neurons via ASIC1a activation. Activation of PVN, the central hub of sympathetic control, induces elevation in blood pressure through the activation of sympathetic nerves (SN). Modified from Sakuta et al. (2016) and Nomura et al. (2019).

Na_x_ is also involved in controlling water intake, mediated by the activation of transient receptor potential vanilloid type 4 (TRPV4) (Figure 7; Sakuta et al., 2016). Originally, TRPV4 was thought to detect extracellular hypotonicity (Liedtke et al., 2000). Later, this group proposed that TRPV4 responds to hypertonicity but not hypotonicity in vivo because the water intake and Fos expression levels induced in OVLT neurons by systemic hypertonicity were lower in *TRPV4*‐KO mice than WT animals (Liedtke & Friedman, 2003). However, the mechanisms by which TRPV4 mediates such responses to hypertonicity have not yet been elucidated.

To directly stimulate the brain's sensing systems, hypertonic solutions were injected into cerebral ventricles, and spontaneous water intake induced immediately after intracerebroventricular injection was measured (Sakuta et al., 2016). Injection of high [Na^+^] artificial CSF (aCSF) induced greater water intake in WT mice than hypertonic aCSF with the same osmolality adjusted using sorbitol, indicating that an increase in CSF [Na^+^] is a more effective stimulus for inducing water intake behavior than CSF osmolality.

Following intracerebroventricular administration of hypertonic NaCl‐containing aCSF, *TRPV4*‐KO, and *Na*_*x*_‐KO mice consumed approximately half the amount of water compared with WT mice (NaCl; Sakuta et al., 2016). Importantly, the water intake in *Na*_*x*_/*TRPV4* double‐KO mice was similar to that in *Na*_*x*_‐KO and *TRPV4*‐KO mice, suggesting a functional overlap in the signaling pathway between Na_x_ and TRPV4.

Subsequently, co‐administration of hypertonic NaCl‐aCSF with arachidonic acid (AA) or 5,6‐epoxyeicosatrienoic acid (EET) increased water intake in *Na*_*x*_‐KO mice, restoring it to levels comparable to those in WT mice, whereas no changes were observed in *TRPV4*‐KO mice. These results strongly support the activation of Na_x_ downstream by EETs in a TRPV4‐dependent manner.

## 9 AUTOANTIBODIES AGAINST Na_X_ AND HYPERNATREMIA

The dysfunction of the Na homeostasis may lead to diseases. Therefore, we searched for cases likely related to the function of Na_x_. Analyses of a pediatric patient with essential hypernatremia revealed the presence of autoantibodies against Na_x_ in the sera (Hiyama et al., 2010). The patient did not have any thirst despite severe hypernatremia. There were no typical elevations in blood antidiuretic hormone (ADH) levels in response to serum hyperosmolality. The patient exhibited a ganglioneuroma primarily composed of Na_x_‐positive Schwann‐like cells. Na_x_ is predominantly expressed in nonmyelinating Schwann cells in the peripheral nervous system. It is presumed that this patient's neoplasia triggered an antitumor immune response. Autoantibodies to Na_x_ likely induce persistent tissue damage within the Na_x_‐positive SFO and OVLT through the activation of complement and infiltration by inflammatory cells. This hypothesis is supported by a passive transfer of the immunoglobulin (Ig) fraction from the patient's serum, which replicated her symptoms in WT mice, causing abnormal reductions in water intake and ADH release. In the sensory CVOs, antibodies easily leak from blood vessels (Broadwell & Sofroniew, 1993) and both the SFO and OVLT have efferent connections with the supraoptic nucleus (SON) and PVN, the sites of ADH production (Mangiapane et al., 1984; Tanaka et al., 1985, 1987; Thrasher et al., 1982). However, it is unlikely that the patient's Ig simply inhibited the function of Na_x_ because *Na*_*x*_‐KO mice do not exhibit defects in ADH release (Nagakura et al., 2010). Analyses of similar cases allowed us to summarize the patients' disorder as adipsic hypernatremia caused by SFO inflammation and dysfunction resulting from autoantibody production against the SFO (Hiyama et al., 2017; Liebrand et al., 2023).

## 10 CONTROLS OF THIRST VIA A NOVEL BRAIN NA SENSOR SLC9A4

Recently, a novel [Na^+^]_o_ sensor involved in thirst induction was identified in the OVLT as SLC9A4, an Na^+^/H^+^ exchanger transporter 4 (Figure 7; Sakuta et al., 2020). Analysis of Na^+^ transport by SLC9A4 revealed an [Na^+^]_o_‐dependent increase, particularly when [Na^+^]_o_ exceeded 150 mM, suggesting its functionality as an [Na^+^]_o_ sensor. Moreover, selective knockdown of OVLT *SLC9A4* using artificial microRNA impaired the water intake following intraventricular infusion of a hypertonic Na solution.

SLC9A4, a nonelectrogenic transporter, does not cause membrane depolarization on its own. However, it is hypothesized that if H^+^ released by the Na^+^/H^+^ exchange transport by SLC9A4 opens acid‐sensitive cation channels in neurons, the neurons are activated. An extracellular [H^+^] increase can be detected by ASIC1a, an Na^+^‐permeable cation channel activated by slight extracellular acidification. Selective inhibition of the acid‐sensitive ion channel ASIC1a by infusion of a hypertonic NaCl solution into the cerebral ventricles impairs the induction of water intake.

Thus, the promotion of drinking behavior in response to an increase in [Na^+^]_o_ involves multiple independent [Na^+^]_o_ sensing mechanisms. Additionally, sensors that detect osmotic pressure, rather than [Na^+^]_o_, may stimulate drinking behavior, a phenomenon that remains to be elucidated.

## 11 Na_X_ IN SALT‐INDUCED ELEVATION IN BLOOD PRESSURE

Excessive Na intake is a risk factor for hypertension, which is traditionally understood to be caused by an increase in blood volume accompanying elevated blood osmotic pressure. Besides this mechanism, excessive salt intake leads to increased [Na^+^]_o_, activating the sympathetic nervous system and consequently raising blood pressure. The [Na^+^]_o_ sensor responsible for this blood pressure regulation is Na_x_ in the OVLT (Figure 7; Nomura et al., 2019).

Experimental models of salt‐induced hypertension involve long‐term ingestion of 2% saline solution instead of water or acute increases in CSF [Na^+^] by intraventricular infusion of hypertonic Na solution. In WT mice subjected to these experiments, significant increases in blood pressure and sympathetic nervous system activity were observed. However, in *Na*_*x*_‐KO mice or mice with localized destruction of the OVLT, the elevation in blood pressure and activation of the sympathetic nervous system were absent.

Within neurons of the OVLT, there exist those that project to the PVN, a central hub for controlling the sympathetic nervous system, which are referred to as OVLT(→PVN) neurons (Nomura et al., 2019). Patch‐clamp experiments conducted on acute brain slices revealed that OVLT(→PVN) neurons exhibited an increased firing frequency in an Na_x_‐dependent manner in response to elevated extracellular [Na^+^]_o_. Optogenetic experiments selectively manipulating neuronal activity in vivo demonstrated that activation of OVLT(→PVN) neurons leads to an increase in blood pressure mediated by the sympathetic nervous system, while inhibition significantly attenuates the blood pressure rise in response to CSF [Na^+^] elevation. This indicates that OVLT(→PVN) neurons mediate the blood pressure increase through the Na_x_ signal. Downstream of the PVN, it has been suggested that the Na_x_ signal is transmitted to the rostral ventrolateral medulla (RVLM), a sympathetic nervous system center in the brainstem.

Through patch‐clamp experiments on acute brain slices and in vivo pharmacological studies, it was determined that the factor responsible for transmitting information from Na_x_‐expressing glias to OVLT(→PVN) neurons is H^+^ (extracellular acidification; Nomura et al., 2019). Similar to the mechanism observed in the SFO, glucose metabolism was found to be involved in the release of H^+^ from Na_x_‐expressing glia. The lactic acid produced within these cells is released extracellularly along with H^+^ through monocarboxylate transporters (MCT), which are the H^+^/lactate cotransporter. These analyses revealed the expression of acid‐sensing ion channel ASIC1a in OVLT(→PVN) neurons. Increased Na^+^ influx causes depolarization thereby activating neurons. Specific inhibition of ASIC1a suppresses sympathetic nervous system‐induced blood pressure elevation in response to Na^+^, and direct injection of an ASIC activator into the OVLT results in sympathetic nervous system‐mediated blood pressure elevation.

Evolutionarily, organisms might have developed such [Na^+^] dependent sympathetic activation mechanisms to elevate blood pressure for the eliminations of sodium through pressure natriuresis. With increased renal pressure, kidneys enhance sodium excretion and reduce extracellular fluid, maintaining normal sodium balance and systemic arterial pressure.

## 12 CONCLUSION

Na^+^ plays a crucial role not only in the generation of action potentials and substance transport via Na‐dependent transporters but also in the regulation of osmotic pressure as an essential constituent of body fluids. Consequently, living organisms have evolved mechanisms to monitor [Na^+^] for the strict maintenance of the [Na^+^] in body fluids. Recent advances in research surrounding the molecular mechanisms underlying Na^+^ sensing in thirst, salt appetite, and blood pressure regulation have opened the door to the development of novel therapeutic strategies for relevant human disorders.

## FUNDING INFORMATION

This study was supported by MEXT/JSPS KAKENHI (grant numbers 18H04055, 21K18269, and 23H00422) and AMED (grant number JP23gm1510001).

## CONFLICT OF INTEREST STATEMENT

The authors declare that they have no conflict of interest.

## ETHICS STATEMENT

Not applicable.
